# Accumulation of Major and Trace Elements in Spider Webs

**DOI:** 10.1007/s11270-015-2369-7

**Published:** 2015-03-20

**Authors:** Justyna Rybak

**Affiliations:** Department of Environmental Protection, Wroclaw University of Technology, Wybrzeże Wyspiańskiego 27, 50-370 Wrocław, Poland

**Keywords:** Agelenidae, Spider webs, Heavy metals, Biomonitoring, Air pollution

## Abstract

The spider webs of *Malthonica ferruginea* (Panzer, 1804) from the Agelenidae family were used for the evaluation of heavy metal contamination, and major and trace elements presence in the air of Wrocław, Poland. The concentrations of 16 elements were determined (Mg, Al, K, Ca, Ti, V, Cr, Mn, Fe, Co, Ni, Cu, Zn, W, Pt, and Pb). Samples of webs were collected from six different locations with low, moderate, and high pollution level (urban of low and high traffic, residential, and postindustrial sites) after 60 days of exposure. Samples collected from high traffic sites and postindustrial site were found to have high contents of elements than residential sites and one of low traffic urban site. The principle component analysis (PCA) and correlation analysis provide important information about the potential sources of the elements in spider webs. Two contamination sources were identified: road traffic emissions and industrial. This was a first-time ever attempt to use webs for biomonitoring of small-scale distribution of airborne major and trace elements in the city of Wrocław.

## Introduction

Major and trace elements, especially heavy metals, are nowadays of much environmental concern. Many anthropogenic sources emit elements into the atmosphere, but vehicular traffic is one of the most harmful hazards to the environment and human health (Harrison and Yin 2000). Different elements behave variously with regard to their vertical distribution pattern, depending on their main form of diffusion, their primary source, etc. Such contaminants, which are transported by air movements, are deposited by wet and dry deposition and can be accumulated on spider webs. The use of mosses, lichens, or vascular plant tissues in sampling has long been shown to be an effective indicator of atmospheric pollution (De Nicola et al. 2008; Boquete et al. 2014; Vuković et al. 2013). But, all of these mentioned biomonitors have some disadvantages. For example, lichens and mosses of similar composition are not easy to find as the differences in its content derive from the growth area. In general, the “bag” technique used for mosses is more relevant as it gives the possibility of comparison with dry and wet deposition, and there are no differences in its composition at the beginning of studies, although the long exposure time and the way of bag construction resulting in its fast dryness could be also limiting factors for this technique (Szczepaniak and Biziuk 2003; Zechmeister et al. 2006). According to Gratani et al. (2008), the basic criteria for the selection of plant biomonitor are that it should occur in large numbers within the studied area, be easy and cheap to sample enabling long-term monitoring of metal pollution in the urban areas (Gratani et al. 2008).

The spider webs, which also capture airborne particulates, are very easy, useful, abundant, and climate-independent indicators of the environment pollution, although their application has been studied only few times (Hose et al. 2002; Rybak 2012; Rybak et al. 2012; Rybak and Olejniczak 2014, Xiao-li et al. 2006). What is more, the application of dust collectors such as impactors requires a constant supervision. Next, it was proved that the use of reference gravimetric method was less effective than the application of mosses in estimating emission of heavy metals from chimneys (Olszowski and Bożym 2014). In contrast to these reference methods, web structure and their all-year-round availability enables an easy and long-term assessment of the air pollution level in a randomly selected places.

Most of the studies in Poland with biomonitors focused on heavy metals content in mosses and lichens tissue (Czarnowska and Gworek 1992; Olszowski and Bożym 2014; Pajak and Jasik 2011). However, there are only a few data on urban environments obtained by this method (Dmuchowski and Bytnerowicz 2009; Samecka-Cymerman et al. 2008.). What is more, the application of webs in urban environment was only studied by Rybak et al. (Rybak 2012; Rybak et al. 2012; Rybak and Olejniczak 2014), but authors focused mainly on organic pollutants and only preliminary studies were done on three heavy metals related to traffic emissions (Zn, Pb, and Pt). Moreover, in the urban environment, naturally growing mosses, lichens, or plants are usually missing, in contrary to spiders who willingly weave their webs in polluted areas which are devoid of sun (e.g., tunnels, road canyons).

The purposes of this study are (i) to determine the levels of Mg, Al, K, Ca, Ti, V, Cr, Mn, Fe, Co, Ni, Cu, Zn, W, Pt, and Pb using spider webs of *Malthonica ferruginea* (Agelenidae) as bioindicators of such pollutants in the city of Wrocław (southwest of Poland) for the first time, and (ii) to identify the most significant factors or source that influences air quality in Wrocław by the principle component analysis (PCA). The obtained results could serve as a baseline information on concentration and pollution sources for future environmental impact assessment. This work introduces a new evaluation method based on web application which is naturally present in the Wrocław landscape.

## Materials and Methods

### Spider and the Web Description

The research was conducted in 2013 at six sites in Wrocław. Species of *M. ferruginea* (Panzer, 1804) from Agelenidae family was chosen for studies because of its web characteristic (dense and large). This reddish, rather common spider with rusty markings on its back occurs in woods, where it may form “colonies” (many webs are found close together). Webs are typically built on the outside of buildings or indoors in unheated sheds, then often in window frames where the light-seeking behavior of insects increase prey availability. The species sometimes inhabits also buildings in open country and in gardens. Adults appear from May to October. The woven web looks like a widespread funnel. The webs often have a tubular retreat (Roberts 1995). They do not eat their own webs when destroyed which is an important feature influencing the level of pollution in the web (Rybak and Olejniczak 2014). The spiders menu could also have the impact on the web composition and pollution level. Spiders from Agelenidae family catch mainly nonflying invertebrates such as beetles, springtails, ants, and earthworms. These preys are not acclimated to overcome long distances like flying insects do. Thus, a contamination of the web originating from prey coming from a distant area is hardly possible in the case of this species (Foelix 1996; Nyffeller et al. 2001; Park and Moon 2002; Rybak and Olejniczak 2014).

All the webs were collected from secluded locations, mainly from buildings, tunnels, or walls protected from weather conditions. Webs were collected within 3 days after 60 days from the creation of the new construction (after the removal an old web). Additionally, in order to get the sufficient sample number, the methods of Champion de Crespigny et al. (2001) were employed to prepare the spider webs for field arrangement (i.e., spiders were bred under laboratory conditions from the juvenile stages, producing their webs in the standard size wooden frames). Such prepared webs were kept away from the pollution (they were closed in the glass, sterile boxes) and then placed to the field at the same day. Webs of similar sizes, age, and weight were used for analyses (Hose et al. 2002).

### Analytical Procedures

Webs were collected into clean glass phials with glass, sterile baguettes, and frozen for further chemical analyses (methodology according Hose et al. 2002; Rybak 2012; Rybak et al. 2012; Rybak and Olejniczak 2014; Xiao-li et al. 2006). Samples were defrosted, dried for 48 h (70 °C), sorted under microscope to remove mechanical residuals, and weighed (approximately 0.3–0.7 g; accuracy 0.0001 g). Digestion system “DK 8 VELP” was used for trace metal analysis. Block (open glass vessels) apparatus allowed for simultaneous digestion of eight samples with “aqua regia” to determine the acid-soluble parts of metals in a deposit. An external temperature controller permitted the conditions for the digestions to be optimized, thus providing the reproducible results. The fumes from the digestion process were removed by a suction system (water jet pump). The concentrations of 16 elements in the webs were assessed by ICP-OES using the sequential selected emission spectrometer ARL 3410 and applying the method of absolute calibration towards standards of Mg, Al, K, Ca, Ti, V, Cr, Mn, Fe, Co, Ni, Cu, Zn, W, Pt, and Pb and for the range of concentrations 0–10 mg/l (0–10 μg/ml) in examined solutions.

Web samples were prepared according to the following procedure: air dry residue 0.2–0.5 g after triple extraction (with 1 ml dichloromethane methanol 9:1 *v*/*v* solution) was digested with the use of nitro-hydrochloric acid (solution of concentrated nitric and hydrochloric acids, 3/1 *v*/*v*) during 24 h, applying triple sequences of the digestion by adding to sample 2 ml of freshly prepared aqua regia (or 1.5 HCl ml + 0.5 HNO_3_ ml). First, mixture was left for 1 h in the room temperature; next, the temperature was slowly increased to avoid upheaval (over 3 h) and kept at boiling temperature during 4 h. Usually at the end of this procedure, approximately 0.5 ml analyte mixture was obtained which was washed and partially dissolved (with 1 % HCl water solution to approx. 5 ml) for the filtration purpose. The final volume of the filtrate attained in 10 ml volumetric flask required for qualitative and quantitative analyses.

The analyses were performed in the Chemical Laboratory of Elemental Analyses in the Chemical Department of the Wrocław University (Rybak 2012; Rybak et al. 2012; Rybak and Olejniczak 2014). Table 1 presents the validation parameters for the method of determining elements in spider webs. All samples were analyzed in batches with blank webs weaved by spiders under laboratory conditions and samples with known metal content (checking the digestion quality and repeatability of standard deviation). As a reference material, pure substances attained from Sigma Aldrich® (analytically pure inorganic compounds) were applied for trace metal analysis usually by mean corresponding metal chlorides.

**Table 1:** Validity check for chemical methods by means of recovery rates (%)

Trace element	Mean (*n* = 3) [μg/g]	SD	RSD [%]	Expected value ± EE [μg/g]	Recovery rate [%]
Mg	225	17.8	7.91	234 ± 12.1	96.1
Al	864	67.2	7.7	840 ± 42	103
K	3616	247	6.83	3200 ± 160	113
Ca	24710	1377	5.57	24310 ± 1215	101.6
Ti	124.3	12.7	10.2	125 ± 6.2	99.4
V	29.3	2.65	9.05	39 ± 1.9	75.05
Cr	51	7.3	14.3	53 ± 2.6	96.2
Mn	253.5	12.7	5.01	250 ± 12.5	101.4
Fe	1657	123.1	7.42	1665 ± 83.2	99.5
Co	1.47	0.23	15.6	1.6 ± 0.08	91.8
Ni	24	3.6	14.9	31 ± 1.5	77.6
Cu	93.6	11.2	12	102 ± 5.1	91.8
Zn	553	34.1	6.17	550 ± 27.5	100.5
W	0.31	0.03	9.67	0.46 ± 0.02	67.4
Pt	0.16	0.03	18.7	0.13 ± 0.006	123
Pb	161.1	12.5	7.75	159 ± 7.9	101

Mean (*n* = 3) element traces, standard deviation SD, and rate of standard deviation RSD (%) determined for web samples with known metal content given in μg/g of not contaminated dry web. Calculated expected value ± estimated 5 % experimental error (EE) comes from web samples with known metal content

### Sampling Sites

**Fig. 1:**
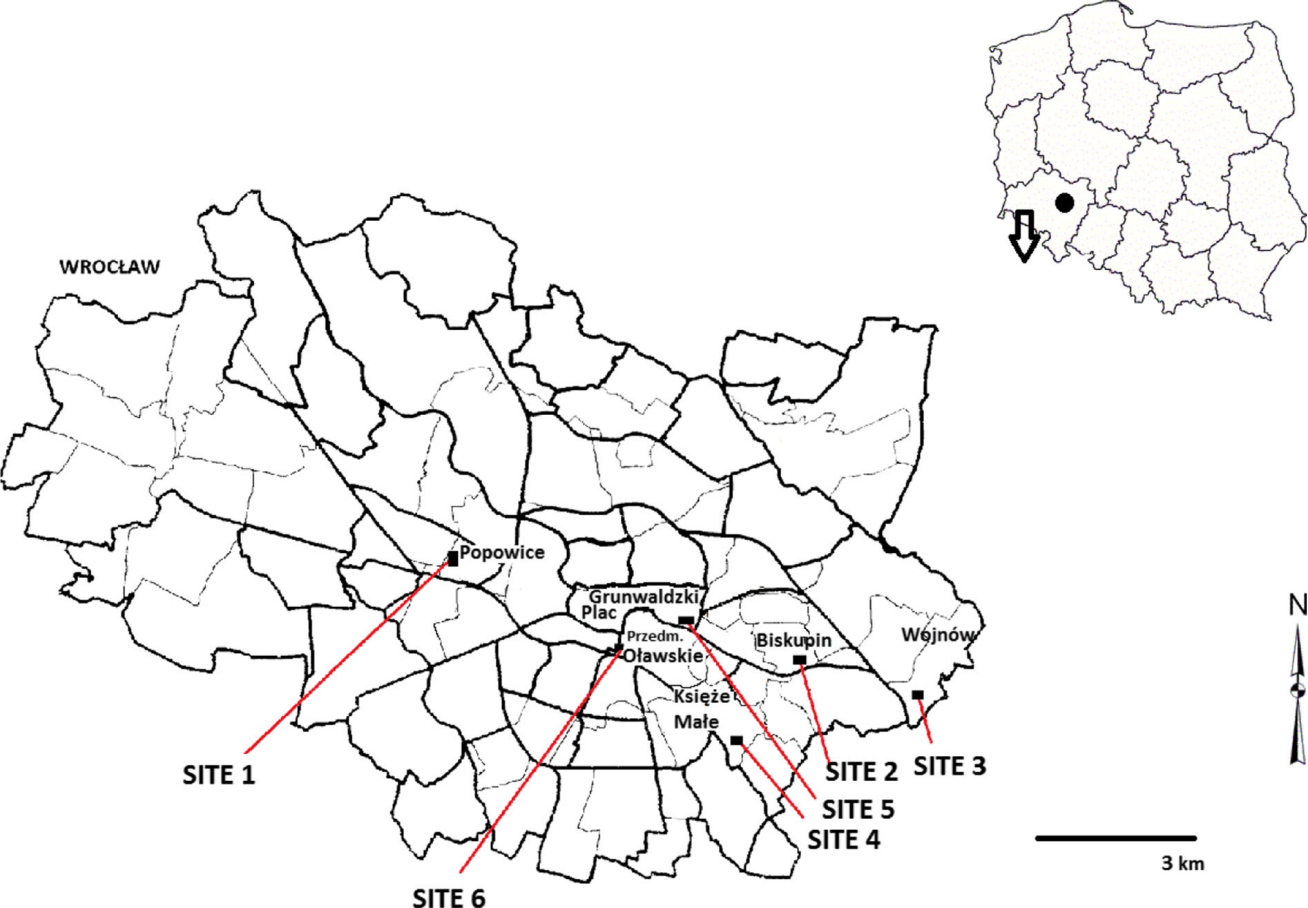
The map of sites selected for studies

The study was carried out in the city of Wrocław, southwest of Poland in 2013. We considered six sites (Popowice, plac Grunwaldzki, Przedmieście Oławskie, Księże Małe, Biskupin, Wojnów). Sites 1 and 2 were situated away from the direct area of high traffic load but in the urban area (urban sites of low traffic); sites 3 and 4 were selected as the residential sites; and sites 5 and 6 were roadsides (Fig. 1). A detailed description of sampling sites is presented in Table 2. All sites were protected from the rain what secured webs before accidental pollutant washing.

**Table 2:** Description of sampling sites

Site	Coordinates for sampling location	Description	The average climatic characteristic and number of collected samples (date of sample collections 11–13, July 2013)
Site 1	N51° 07′ 26.32″/E17° 00′ 01.31″ Popowice urban site of low traffic	A small tunnel linking two blocks of flats, surrounded by allotments with low traffic intensity.	Maximum daily temperature 22.3 °C. Minimum daily temperature 12.3 °C. The average wind speed 1.7 m/s (*n* = 7)
Site 2	N51° 5′ 58.31″ N/E17° 5′ 36.74″ E Biskupin urban site of low traffic	A quiet area with low buildings and moderate traffic intensity surrounded by park and Odra river.	Maximum daily temperature 22.5 °C. Minimum daily temperature 11.2 °C. The average wind speed 2.9 m/s (*n* = 9)
Site 3	N51° 5′ 50.77″/E17° 9′ 28.44″ Wojnów residential area	A quiet area with low and buildings traffic intensity surrounded by woodlands and Odra river.	Maximum daily temperature 21 °C. Minimum daily temperature 12.6 °C. The average wind speed 2.8 m/s (*n* = 14)
Site 4	N51° 4′ 14.01″/E17° 8′ 1.17″ Księże Małe/Siechnice residential area	The meadows surrounded by the system of ditches and channels with low traffic intensity. A drinking water is delivered for the city of Wrocław from this area. This area is bordering on former smelter processed chromite ores closed in the mid-1990s. As a result of its activity a huge chromium-iron heap was left, posing the threat to local environment.	Maximum daily temperature 21.3 °C. Minimum daily temperature 12.1 °C. The average wind speed 2.2 m/s (*n* = 11)
Site 5	N51° 6′ 38.32″/E17° 3′ 28.66″ E Plac Grunwaldzki roadside	This centrally located square is a main interchange with heavy motor traffic 24/7.	Maximum daily temperature 22.7 °C. Minimum daily temperature 14.3 °C. The average wind speed 1.2 m/s (*n* = 10)
Site 6	N51° 5′ 51.31″/E17° 2′ 34.85″ Przedmieście Oławskieroadside	One of main, central and very important communication hubs in the city of Wrocław with heavy motor traffic 24/7.	Maximum daily temperature 23.2 °C. Minimum daily temperature 14.1 °C. The average wind speed 1.4 m/s (*n* = 6)

### Statistical Analysis

The data were processed by statistical tests, using a STATISTICA® software package. Data were checked for normal distribution (Shapiro–Wilk’s *W*-test) and homogeneity of variance (Levene’s test). Tests of significance were made at 95 % confidence level. The significance of differences in mean contaminant concentrations between site types was performed by means of Kruskal–Wallis ANOVA on ranks for non-normally distributed data. Spearman’s rank correlation coefficients were calculated on elemental concentrations for all sites (samples). Correlations were considered as strong when higher than 0.7, and *p* values were 0.05.

Principal component analysis (PCA) with varimax rotation was successfully employed to identify probable air pollution sources contributing to the variation of air quality in the city of Wrocław.

The enrichment factor (EF) was computed in relation to the concentrations of Ti at site 3 of lowest pollution according to the formula EF = (*c*_E_/*c*_Ti_)_site *n*_/(*c*_E_/*c*_Ti_)_site 3_.

## Results and Discussion

### Elements Concentrations in Webs

The web samples from six sites revealed varying concentrations of all 16 elements (Mg, Al, K, Ca, Ti, V, Cr, Mn, Fe, Co, Ni, Cu, Zn, W, Pt, and Pb) (Table 3). According to the values, the most accumulated element in webs was Ca, then in descending order Fe > K > Al > Zn > Mg > Ti > Mn > Cu > Pb > Ni > Cr > V > Co > Pt > W. The greatest differences between residential site (site 3) and roadside (site 5) were observed for Ca with concentration nearly 58-fold higher at roadside than residential site (Table 3). Webs showed lower element concentrations (Al, K, V, Cr, Fe, Ni, Zn, Cu, W, Pt, Pb) at one residential site (site 3) and urban sites of low traffic intensity (sites 1, 2) than centrally located urban sites (roadsides) and postindustrial and residential sites (sites 4, 5, and 6) (Tables 3 and 4). Surprisingly, site 2, Biskupin (urban site of low traffic), although located in a quiet area showed higher concentrations of Al, K, Ca, Ti, V, Co, Cr, Ni, Cu, and Pt than the two other low traffic sites (urban site 1 of low traffic and residential site 3) which is probably connected with the dominance of southwestern winds in Wrocław (Dubicki et al. 2002); as a result, road dust suspends more in this area.

**Table 3:** Major and trace elements concentration values (*n* = 57) for web samples collected at six different sites in Wrocław city (mean value ± S. D.) Trace element

	Site 1 (urban site of low traffic)	Site 2 (urban site of low traffic)	Site 3 (residential site)	Site 4 (residential site)	Site 5 (roadside)	Site 6 (roadside)
Mg	82.1 ± 23.8	73.5 ± 11.2	266.1 ± 11.4	301.6 ± 87.1	201.5 ± 11.5	1139 ± 261
Al	710 ± 36.5	1188 ± 95.2	372.1 ± 18.5	1996 ± 160	3727 ± 34.4	2057 ± 39.1
K	3309 ± 10.7	3089 ± 90.7	574.2 ± 12.3	5325 ± 13.3	16,028 ± 149	6079 ± 21.1
Ca	2879 ± 159	13,752 ± 153	1052 ± 18.5	7762 ± 45.06	60,213 ± 186	24,288 ± 143
Ti	158.4 ± 24.8	302.06 ± 28.7	106.3 ± 12.9	255.7 ± 29.6	634.01 ± 61.9	638.8 ± 57.6
V	5.82 ± 1.77	13.3 ± 1.2	1.56 ± 0.05	9.87 ± 2.68	28.06 ± 7.62	17.9 ± 1.05
Cr	1.17 ± 1.75	3.54 ± 1.68	1.3 ± 1.04	68.2 ± 11.8	38.7 ± 7.2	29.6 ± 4.27
Mn	212.7 ± 38.6	218.5 ± 36.3	256.8 ± 15.5	98.8 ± 34.6	660.7 ± 10.5	305.4 ± 10.5
Fe	1399 ± 155	953.7 ± 292	2382 ± 180	7774 ± 20	9654 ± 867	2533 ± 1528
Co	1.03 ± 0.36	1.4 ± 0.2	0.4 ± 0.14	1.2 ± 1.19	3.7 ± 1.1	2.63 ± 1
Ni	21 ± 1.8	19.7 ± 2.23	3.4 ± 0.23	23.3 ± 1.78	46.6 ± 12.5	34.4 ± 6.6
Cu	80.6 ± 8.16	69.8 ± 11.9	12.2 ± 1.7	124.1 ± 8.36	796.9 ± 78.4	225.01 ± 7.94
Zn	489.2 ± 31.5	321.5 ± 16.7	90.6 ± 12.2	3268 ± 8.9	2833 ± 176	1858 ± 39.1
W	0.71 ± 0.07	0.8 ± 0.15	0.64 ± 0.08	0.33 ± 0.13	1.72 ± 1.004	1.05 ± 0.03
Pt	n.d.	0.41 ± 0.002	n.d.	4.76 ± 0.2	0.27 ± 0.001	2.6 ± 0.2
Pb	94.3 ± 11.7	70.8 ± 1.38	15.04 ± 1.08	121.23 ± 3.71	525.6 ± 9.59	212.8 ± 55.9
Total	9445 ± 511.3	20,080 ± 752	5136 ± 288	27,140 ± 435	95,395 ± 1682	39,430 ± 2180

Concentrations of elements are given in μg/g of dry weight

*n.d.* not detected

**Table 4:** Results of Kruskal–Wallis ANOVA on ranks

Element	*H* values	Observed *p* values
Mg	0.17	0.75
Al	5.75	0.001*
K	3.55	0.03*
Ca	0.38	0.53
Ti	0.43	0.6
V	3.66	0.01*
Cr	3.28	0.04*
Mn	1.33	0.25
Fe	3.74	0.02*
Co	4.54	0.32
Ni	6.01	0.002*
Cu	3.71	0.02*
Zn	7.1	0.007*
W	6.18	0.01*
Pt	7.57	0.009*
Pb	3.83	0.02*

*Significant differences among samples collected at sites 1, 2, and 3 and sites 4, 5, and 6 (at *p* values <0.05)

Enrichment factors (EF) comparing concentrations of Ti at site 3 to other elements at studied sites are shown in Table 5. The highest EFs at site 1 were found for Cu (4.43), Pb (4.2), Ni (4.14), at site 2 for Ca (4.59), V (2.99), Ni (2.04), and Cu (2.01); at site 4 for Cr (21.7), Zn (14.9), and Cu (4.23); at site 5 for Cu (10.9), Ca (9.58), Pb (5.85), and Zn (5.24); and at site 6 for Ca (3.83), Cr (3.79), Zn (3.41), and Pb (2.35). The samples from sites 4 and 5 showed the highest enrichment factors for Cr, Zn, Cu, and Ca (see Table 5). They were obtained probably due to high traffic density (site 5) and industrial pollution, although an unknown fraction of deposited substances may be lost with precipitation, because webs were protected from the leaching.

**Table 5:** Enrichment factor (EF) according concentration of Ti at site 3 to concentrations of other elements at studied sites

E.F./site	Site 1	Site 2	Site 4	Site 5	Site 6
Mg	0.2	0.09	0.47	0.12	0.71
Al	1.3	1.12	2.22	1.67	0.92
K	3.86	1.9	3.85	4.67	1.76
Ca	1.83	4.59	3.06	9.58	3.83
V	2.5	2.99	2.62	3.01	1.91
Cr	0.6	0.95	21.7	4.99	3.79
Mn	0.55	0.29	0.16	0.43	0.19
Fe	0.4	0.14	1.35	0.67	0.17
Co	1.72	1.23	1.24	1.55	1.09
Ni	4.14	2.04	2.85	2.29	1.68
Cu	4.43	2.01	4.23	10.9	3.07
Zn	3.62	1.24	14.9	5.24	3.41
W	0.74	0.43	0.21	0.45	0.27
Pt	–	–	–	–	–
Pb	4.2	1.65	3.34	5.85	2.35

Emissions from the vehicles mean the emission from the combustion systems, as well as mechanical usage of car elements and road surface. Such elements as Zn, Cu, Pb, Pt, Fe, and Cr enter the atmosphere as a result of usage of brakes system, disc, or catalytic converter and the detrition of tires (Amato et al. 2010; Gugamsetty et al. 2012).

#### Zinc

Zinc is always connected with anthropogenic emission sources such as traffic emissions. It is transmitted (altogether with Ca) as a result of engine oil usage. According to Goix et al. (2013), Zn was proposed as a new tracer of vehicle emissions since banning the lead presence in fuel as it occurs in fuel, engine lubricants and anti-wear additives (Pulles et al. 2012). The maximum value of Zn was measured at site 4 (3268 ± 8.9 μg/g; Table 3) then at site 5 (2833 ± 176 μg/g; Table 3). Postindustrial site 4 (residential site) was described by the Regional Inspectorate for Environmental Protection (Wojewódzki Inspektorat Ochrony Środowiska) as highly polluted (in zinc and other elements such as chromium and iron), and zinc values recorded there in soil samples were higher than average in Wrocław (WIOŚ 2012), thus the high values of this element probably originate from this source. Site 5 (roadside) is characterized by high frequency of heavy vehicle traffic. The high values of Zn derive probably from vehicle emissions.

#### Copper

Brake wear from road traffic vehicles is an important source of atmospheric (particulate) copper concentrations in Europe (Hulskotte et al. 2007). The maximum value was reached also at heavy traffic site 5 (796.9 ± 78.4 μg/g; Table 3).

#### Lead

The use of unleaded gasoline has decreased significantly the release of tetraethyl lead into the air but the overall lead presence in the air is still high. Also, in our studies, the high values were observed near the major roads (Moore et al. 2011). The maximum value was reached 525.6 ± 9.59 μg/g (Table 3) at site 5 (roadside) too which is known for high frequency of heavy vehicle traffic.

#### Platinum

A pollution control device fitted to cars and lorries, the catalytic converter (autocatalyst) is the largest application of platinum group metals. In our studies, platinum was mainly found at traffic related sites (sites 5 and 6, both roadsides) but also at postindustrial residential site 4 where reached the maximum level in webs (4.76 ± 0.2 μg/g; Table 3). Although, the low traffic intensity was observed there, surroundings of the heap were characterized by higher concentrations of Zn, Cu, and Cr in soil (WIOŚ 2012). The measurements taken by the Regional Inspectorate for Environmental Protection (WIOŚ) were focused on selected elements only (Zn, Pb, Cd, Cr, Cu, Hg, Ni, and As). On the other hand, our previous studies also recorded the relatively high level of platinum content in this area (Rybak 2012).

#### Chromium

Chromium is used in steel presented in vehicles, and also car cylinders are usually covered with layer of chromium. This element is also used in paper mills and paints. The highest value of Cr was obtained at postindustrial residential site 4 (68.2 ± 11.8 μg/g; Table 3), then, as previously, at high traffic site 5 (38.7 ± 7.2 μg/g; Table 3). The high value reached at postindustrial site 4 is probably connected with the presence of the slag heap as this type of waste was left after the activity of closed smelter. High values of this metal were also reported here in soil samples by Environmental Pollution Agency WIOŚ (WIOŚ 2012).

#### Iron

Iron is the most widely used of all the metals. Its low cost and high strength make it indispensable in engineering applications such as the construction of machinery and also vehicles (Moore et al. 2011). The maximum value was reached also at high traffic site 5 (9654 ± 867 μg/g; Table 3), then at postindustrial residential site 4 (7774 ± 20 μg/g; Table 3) which is again directly connected with the presence of the slag heap.

#### Manganese, Nickel, and Vanadium

The presence of Mn, Ni, and V derives from the petrol combustion (Moore et al. 2011). Vehicle traffic is responsible for the emission of some small quantities of other metals, like nickel. Nickel originates from coal, oil, and gas combustion. The maximum value was measured at high traffic site 5 (46.6 ± 12.5 μg/g; Table 3). Manganese is added instead of Pb to increase the octane number of fuel. The highest value of Mn was obtained also at site 5 (660.7 ± 10.5 μg/g; Table 3). Vanadium is often associated with lead as a component of gas oil and coal. It is also used in industries during nickel-vanadium steel production. The maximum value was reached also at high traffic site 5 (28.06 ± 7.62 μg/g; Table 3) which is probably due to the combustion of oil and other fuels.

#### Other Elements

Such elements like Mg, Al, Ti, K, Ca, Co, and W could originate mainly from dispersed road windblown dust (Szczepaniak and Biziuk 2003), but some of them (Co, Al, Ca) could be also emitted by road traffic (Zechmeister et al. 2006). Cobalt is used to produce alloys used in the manufacture of engines. Cobalt compounds are also used to color paints and used as a drier for porcelain enamel and paints. Al came from car body and Ca is attributed to brake wear and lubricating oil (Zechmeister et al. 2006). All these three elements (Co, Al, Ca) reached their maxima at high traffic site 5 (3.7 ± 1.1 μg/g for Co, 3727 ± 34.4 μg/g for Al, 60,213 ± 186 μg/g for Ca; Table 3).

In general, the total concentration of all elements varied from 5136 μg/g (dry weight) assessed at site 3 (residential site) to 95,395 μg/g (d.w.) at site 5 (urban site) (Table 3). Thus, we could assume the airborne particles comprise about, from 10 mg (1 %) to 200 mg (20 %) of the total weight of web depending on the site (we took into consideration the total concentration of all elements and added similar value for other pollutants presented in dust such as SiO_2_ not studied in this paper).

### Comparison to Literature Values

Comparison with literature values was difficult because of the limited numbers of studies and elements considered in previous studies concerning webs (Hose et al. 2002; Xiao-li et al. 2006). Xiao-li et al. (2006) studied only four heavy metals (Pb, Zn, Cu, and Cd) in webs of *Achaearanea tepidariorum* and *Araneus ventricosus*. The highest values they recorded were 289.74 ± 134.02 μg/g dry weight for Pb, 647.64 ± 280.79 μg/g dry weight for Zn, and 17.35 ± 4.14 μg/g dry weight for Cu after 7 days of web exposure in the studied area in China. Pb, Zn, and Cu concentrations in the present study (525.6 ± 9.59 μg/g for Pb, high traffic site 5; 3268 ± 8.9 μg/g for Zn, postindustrial residential site 4; 796.9 ± 78.4 μg/g for Cu, high traffic site 5; Table 3) were nearly 2- to 50-fold higher than recorded by Xiao-li et al. (2006), but webs in this study were exposed much longer (for 60 days).

Hose et al. (2002) studied two heavy metals (Zn and Pb) in webs of *Badumna socialis* and *Stiphidion facetum* in Australia. They obtained respectively 1400 μg/g for Pb and 800 μg/g for Zn (results were presented in charts form) for webs collected “between March and September 1994”; thus, they were probably exposed to pollution for 5–6 months (three times longer than in present studies). Pb concentrations in webs of *M. ferruginea* were in the range of those measured in webs of Australian spiders (three times lower) (Hose et al. 2002). Zn concentrations were similar, but the exposure time of Hose et al. (2002) studies was much longer, thus we got higher concentrations of Zn which is probably linked to industrial activity in the studied region (postindustrial site 4 near slag heap).

### Deposition of Elements

Emitted metals are mainly transported in the atmosphere in the particulate phase. Most particles >10 μm are deposited gravitationally, while finer particles are usually deposited by atmospheric precipitation, such as snow or rain (Miler & Gosar 2013). According to Blagnytė & Paliulis (2010), air pollutants are deposited on mosses in aqueous solution, in gaseous form or attached to particles. A high proportion of the pollutant load accumulates in mosses through wet deposition. The attachment of particles in mosses is affected by the size of the particles and the surface structure of the mosses (Blagnytė & Paliulis 2010). As the webs were protected from the rain, only humid could contribute to dry deposition. Further, river or pond–spray aerosols (sites 1, 2, 3, 4, and 5 were situated near watercourses) may contribute of total trace element emissions to the atmosphere. In general, we can assume that webs mainly trap solid aerosols. Particles >10 μm probably predominate on the surface of the web. In the paper published by Ortega-Jimenez & Dudley (2013), the authors suggest the capture success of spider webs is linked to its microstructure and surface charge, ornamentation, and wind-induced vibrations. The positively charged droplets of water are attracted to the negatively charged webs. The smaller the droplets are, the better attraction is achieved which suggests the dust particles can also behave in the same way on the web (Rybak & Olejniczak 2014). The webs surface size is also a very important feature for accumulation of elements.

As the metals mostly occur in solid forms, when assessing variations in quantities and types of metal-bearing particles and their source, SEM/EDS techniques should be applied in the future studies.

### Possible Sources of Air Pollution

Spearman’s rank correlation coefficient values are presented in Table 6. Strong correlations (0.7) were observed between Al and K, Ca, and Cu, and between Ti and V which are typical pattern for such lithogenic elements. Strong correlations were also recorded between V and Cr, Ni and Cu, between Cr and Fe, Ni, and Zn, between Mn and Fe, between Ni and Cu and Pb, and between Zn and Pb which could be related with traffic pollution and the presence of irony-chromium slag heap (postindustrial site 4).

**Table 6:** Spearman rank correlation matrix on all elemental concentrations in webs (sites 1, 2, 3, 4, 5, 6)

	Mg	Al	K	Ca	Ti	V	Cr	Mn	Fe	Co	Ni	Cu	Zn	W	Pt	Pb
Mg																
Al	0.08															
K	−0.06	*0.9*														
Ca	−0.33	*0.95*	*0.63*													
Ti	0.11	*0.68*	0.47	0.37												
V	0.07	*0.66*	0.34	0.32	*0.75*											
Cr	−0.05	0.36	0.42	0.19	0.25	*0.7*										
Mn	−0.2	0.29	0.45	*0.54*	0.28	0.34	0.46									
Fe	−0.06	*0.56*	0.42	−0.03	0.46	0.46	*0.74*	*0.9*								
Co	−0.09	*0.68*	*0.56*	0.29	*0.69*	0.45	0.35	0.21	0.34							
Ni	0.05	*0.67*	0.27	*0.62*	0.46	*0.77*	*0.75*	*0.51*	*0.5*	0.49						
Cu	0.08	*0.78*	0.34	0.46	0.35	*0.79*	*0.64*	0.44	*0.59*	0.24	*0.87*					
Zn	0.009	0.31	0.47	0.32	0.23	*0.55*	*0.87*	0.1	*0.77*	0.37	*0.64*	*0.83*				
W	0.3	0.45	0.29	0.13	0.35	0.45	*0.56*	−0.35	−0.43	0.29	0.15	0.29	0.42			
Pt	0.33	0.35	0.1	−0.27	0.27	−0.14	0.49	−*0.65*	−*0.54*	0.35	−0.09	0.03	0.46	0.36		
Pb	−0.05	*0.63*	0.49	*0.54*	0.44	*0.66*	*0.5*	*0.51*	*0.6*	*0.62*	*0.76*	*0.78*	*0.89*	0.02	−0.08	

Correlation above 0.5 are presented in italics

The principal component analysis (PCA), considering only the data set of polluted sites (sites 4, 5, and 6), has successfully extracted two rotated principal components which illustrate the sources of air pollution in Wrocław (Table 7).

**Table 7:** Factor loadings for varimax rotated PCA of elements data in webs (sites 4, 5, 6). Platinum was excluded

	Factor 1	Factor 2
V	*0.93*	0.29
Cr	0.07	*0.91*
Mn	*0.96*	0.23
Fe	0.65	*0.7*
Ni	*0.93*	0.29
Cu	*0.91*	0.35
Zn	0.24	*0.81*
Pt	−0.59	0.31
Pb	*0.92*	0.32
Initial Eigenvalue	6.27	1.52
Variance (%) Cumulative (%)	69.73 69.73	16.92 86.66

Italicized figures are essential to illustrate particular parameters contributing to component variability (>0.7)

**Fig. 2:**
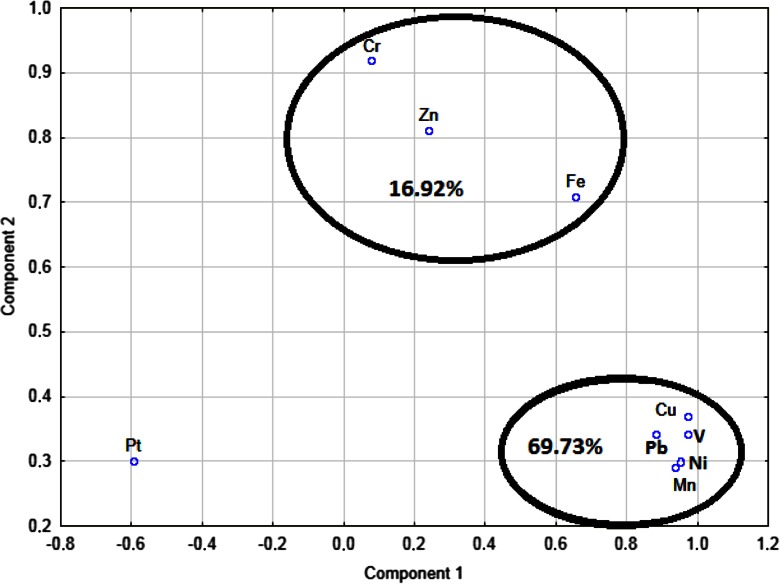
PCA analysis for sites 4, 5, and 6 excluding Mg, Al, K, Ca, Ti, Co, and W. Cumulative percentage of variation explained by two components 86.66 %. Webs of species *M. ferruginea* were used

Considering the data set of urban traffic related sites and postindustrial sites (sites 4, 5, and 6), two components were identified explaining 86.66 % of the variance (Table 7, Fig. 2). The first component (factor 1) accounted for 69.73 % of the total variance and was dominated by V, Mn, Ni, Cu, and Pb. This component is therefore probably attributed to traffic pollution. Moreover, all these elements could also originate from dispersed road dust. The second factor (factor 2) accounted for 16.92 % of the total variance, and it was significantly correlated to Cr, Fe, and Zn high factor loadings (Table 7 and Fig. 2). Thus, it was attributed to the presence of slag heap and high content of Zn, Fe, and Cr in soil near the site 4.

The principal component analysis is a common tool for the identification of sources of metal concentrations (Table 7, Fig. 2). Pt is clearly separated from the rest of the elements and usually originates from anthropogenic sources. This element showed no correlation with other elements (Table 6). Although, its isolated position in the PCA remains unclear. Component 1 dominated by V, Mn, Ni, Cu, and Pb is of traffic origin. The close correlation of Cu and Ni, V and Cu, V and Ni, and Pb and Ni (Table 6) can also be explained by its anthropogenic origin. These elements usually reflect usage of brakes system, catalytic converter, the detrition of tires, or petrol combustion (Gugamsetty et al. 2012; Moore et al. 2011). Component 2 is dominated by Cr, Zn, and Fe and possibly represents industrial sources. The close correlation of Zn and Cr, Zn and Fe, and Cr and Fe (Table 6) can be also explained by their common source. What is more, Zn and Cn have the highest enrichment factors (Table 5). The irony-chromium slag heap in the area of site 4 probably had the main influence on the heavy metal distribution.

## Conclusions

In general, Wrocław represents an example of city where air pollution is high, causing risk to people health. Air pollution is most likely due to emissions from motor vehicles, contamination, and comes also from local sources mostly industrial.

In this study, we determined the environmental quality of six sites in terms of major and trace metal accumulation in spider webs. The method provided first values on deposition of 16 elements with the use of spider webs. Webs of *M. ferruginea* appeared to be very sensitive tool to asses spatial distribution of contaminants. Principal component analysis summarizes the dataset into two major components representing different sources of the elements. Traffic pollution may be responsible for the observed association of V, Mn, Ni, Cu, and Pb in PC 1 at sites 5 and 6 (roadsides). PC 2 with high loadings of Zn, Fe, and Cr is attributed to other sources such as presence of slag heap produced by former smelter processing chromite ores at site 4 (postindustrial residential site).

The results of this study demonstrate the usefulness of spider webs for studying major and trace element emissions and depositions deriving from different sources as industries and traffic. Tiny structure of webs creates ideal net functioning as a trap for road windblown dust and atmospheric aerosols. This is a new, cost-efficient, and easy method of biomonitoring which allows to get as good results as obtained with mosses and plants. Although, present studies could only be qualitatively compared with research carried out with other matrices such as mosses. Indeed, disparate matrices and supposedly unrelated contamination levels make impossible direct quantitative comparison.

The main advantage of usefulness of spider webs is that webs are widespread, providing the high density of sampling sites, enabling long-term monitoring of pollution level in the selected area. Our study recommend the use of *M. ferruginea* webs as this species is naturally present and widely distributed, additionally it can be easily bred under laboratory conditions in order to use its webs as the future monitoring tool.
